# Aptamer-Based Proteomics Identifies Mortality-Associated Serum Biomarkers in Dialysis-Dependent AKI Patients

**DOI:** 10.1016/j.ekir.2018.04.012

**Published:** 2018-05-03

**Authors:** Li-Rong Yu, Jinchun Sun, Jaclyn R. Daniels, Zhijun Cao, Laura Schnackenberg, Devasmita Choudhury, Paul M. Palevsky, Jennie Z. Ma, Richard D. Beger, Didier Portilla

**Affiliations:** 1 Division of Systems Biology, National Center for Toxicological Research, U.S. Food and Drug Administration, Jefferson, Arkansas, USA; 2 Division of Nephrology, Center for Immunity, Inflammation and Regenerative Medicine, University of Virginia, Charlottesville, Virginia, USA; 3 Salem Veterans Affairs Medical Center, Salem, Virginia, USA; 4 VA Pittsburgh Healthcare System, University of Pittsburgh, Pennsylvania, USA

**Keywords:** acute kidney injury, aptamers, biomarkers

## Abstract

**Introduction:**

Currently, no effective therapies exist to reduce the high mortality associated with dialysis-dependent acute kidney injury (AKI-D). Serum biomarkers may be useful in understanding the pathophysiological processes involved with AKI and the severity of injury, and point to novel therapeutic targets.

**Methods:**

Study day 1 serum samples from 100 patients and day 8 samples from 107 patients enrolled in the Veteran’s Affairs/National Institutes of Health Acute Renal Failure Trial Network study were analyzed by the slow off-rate modified aptamers scan proteomic platform to profile 1305 proteins in each sample. Patients in each cohort were classified into tertiles based on baseline biomarker measurements. Cox regression analyses were performed to examine the relationships between serum levels of each biomarker and mortality.

**Results:**

Changes in the serum levels of 54 proteins, 33 of which increased and 21 of which decreased, were detected when comparing samples of patients who died in the first 8 days versus patients who survived >8 days. Among the 33 proteins that increased, higher serum levels of fibroblast growth factor-23 (FGF23), tissue plasminogen activator (tPA), neutrophil collagenase (matrix metalloproteinase-8), and soluble urokinase plasminogen activator receptor, when stratified by tertiles, were associated with higher mortality. The association with mortality persisted for each of these proteins after adjusting for other potential risk factors, including age, sex, cardiovascular sequential organ failure assessment score, congestive heart failure, and presence of diabetes. Upper tertile levels of FGF23, tPA, and interleukin-6 on day 8 were associated with increased mortality; however, FGF23 barely lost significance after multivariable adjustment.

**Conclusions:**

Our results underscore an emerging proteomics tool capable of identifying low-abundance serum proteins important not only in the pathogenesis of AKI-D, but which is also helpful in discriminating AKI-D patients with high mortality.

Acute kidney injury requiring dialysis (AKI-D), one of the most serious complications for hospitalized patients, is associated with prolonged length of stay, mortality, and progressive chronic kidney disease (CKD) among survivors.^1^, ^2^, ^3^, ^4^ Better understanding of the pathogenesis of AKI-D and risk factors associated with mortality is needed. Previous studies have examined the role of urine and serum biomarkers to predict AKI severity. The Biologic Markers of Recovery for the Kidney study conducted in AKI-D patients found that decreasing urine levels of hepatocyte growth factor and neutrophil gelatinase−associated lipocalin predicted recovery.^5^, ^6^, ^7^ In this study, higher serum levels of tumor necrosis factor receptor 1 and interleukin (IL)-8 were associated with reduced dialysis-free survival at 60 days.^6^, ^7^ These studies suggested that serum AKI biomarkers could provide additional prognostic information, although they might not have established a direct relationship between the degree of kidney injury and increased mortality.^8^

Mass spectrometry (MS) and affinity multiplexing assays have been used for proteomic biomarker discovery. MS-based proteome profiling is ideal for discovery of novel biomarkers but lacks throughput in the validation phase when hundreds to thousands of samples require analysis.^9^ Affinity proteome profiling with high multiplexing capabilities has emerged as a more efficient method for biomarker studies. Affinity-based protein profiling uses antibody bead arrays with >4000 antibodies.^10^ Recently, a new proteomics platform, slow off-rate modified aptamers (SOMAmers) scan, was developed for the examination of global protein expression.^11^, ^12^ SOMAmers rely on the natural 3-dimensional folding of single-stranded DNA-based protein affinity reagents. SOMAmers are deoxyoligonucleotides with unique intramolecular motifs that bind to the respective protein targets in native conformations.^13^ The technology enables a simultaneous quantitative analysis of 1305 proteins per sample using small sample volumes. SOMAscan has been applied to studies on several types of medical conditions to identify proteins and molecular mechanisms involved in disease progression.^14^, ^15^, ^16^, ^17^, ^18^, ^19^ To better understand the pathogenesis associated with high mortality in AKI-D patients, we performed SOMAscan proteomic profiles on serum samples collected from 207 participants in the Veterans Affairs/National Institutes of Health Acute Renal Failure Trial Network (ATN) study.

## Concise Methods

### Study Design

The ATN study was a prospective, multicenter randomized clinical trial that evaluated the intensity of renal replacement therapy in critically ill patients with AKI-D; it enrolled 1124 patients from 27 Veterans Affairs and 12 academic medical centers across the United States. Outcomes included 60-day mortality, recovery of kidney function, and intensive care unit and hospital length of stay. Details of the study protocol, including inclusion and exclusion criteria, have been previously published.^20^, ^21^ Patients enrolled in the ATN study were critically ill adults (18 years or older) who had AKI that was clinically consistent with acute tubular necrosis and failure of ≥1 nonrenal organ (defined as a nonrenal sequential organ failure assessment [SOFA] score of ≥2) or sepsis. Consent for sample collection and at least 1 sample were obtained from 827 of the 1124 subjects who participated in the ATN study. A total of 819 patients provided samples on day 1 and 573 patients on day 8, with 565 patients contributing samples on both day 1 and day 8. For this *post hoc* analysis, we randomly selected 100 day 1 serum samples from patients who either died before (n = 49) or survived to (n = 51) day 8 and 107 day 8 serum samples from patients who either died before (n = 24) or survived to (n = 83) day 28. Clinical data available through the National Institute of Diabetes and Digestive and Kidney Diseases (NIDDK) data repository via a cross-walk file was then linked to the de-identified samples analyzed for this study. This *post hoc* analysis was approved by the Salem Veteran’s Affair Medical Center and Food and Drug Administration Institutional Review Boards.

### SOMAscan Proteomic Profiling

We performed quantitative proteome profiling of AKI samples using the SOMAscan assay developed by Somalogic Inc. (Boulder, CO) as described previously.^11^, ^22^ A total of 1305 serum proteins were measured simultaneously in a single assay using 50 μl of sample per the manufacturer’s sample processing procedures. In each assay, 6 calibrator samples, 2 quality controls (QCs), and 1 blank sample from the assay manufacturer were included. We detected the fluorescence signal intensities of SOMAmers using an Agilent microarray scanner (Agilent Technologies, Inc., Santa Clara, CA).

### Data Processing

We used SOMAscan standard calibration and normalization procedures, including hybridization control normalization, median signal normalization, and between-run calibration, to remove systematic biases. The hybridization control normalization was achieved using the global relative fluorescence units (RFUs) of each of the 12 hybridization control sequences. For median signal normalization, a scale factor was derived for each dilution set. Between-run calibration was achieved using the 6 calibrators from common pooled samples. After normalization and calibration, we stratified the day 1 samples based on survival to day 8 and stratified day 8 samples based on survival to day 28. Welch’s *t*-test was performed for logarithm RFUs to find significantly changed proteins between the deceased and surviving groups. The *t*-test *P* values were also adjusted for this multiplex assay to calculate the false discovery rate (FDR) using the Benjamini and Hochberg method.^23^

### Ingenuity Pathway Analyses

We used Ingenuity Pathway Analyses (IPA) software (QIAGEN, Redwood City, CA) for gene ontology, pathway analyses, and core analysis comparison for the proteins that changed significantly at day 1 in patients who died in <8 days versus those who survived >8 days. A *P* value of <0.05 was considered significant.

### Enzyme-Linked Immunosorbent Assay of Fibroblast Growth Factor 23

To correlate fibroblast growth factor-23 (FGF23) SOMAscan intensities with serum levels of FGF23 we used human FGF23 (C-terminal) enzyme-linked immunosorbent (ELISA) kits (Immutopics, Inc., San Clemente, CA). We randomly selected 30 samples from day 1 and 31 samples from day 8 cohorts with SOMAscan RFUs ranging from the lowest to highest. ELISA was performed in duplicate per manufacturer recommendations. Correlation between SOMAscan and ELISA data was assessed using multivariate correlation analysis under JMP version 12 (SAS Institute Inc., Cary, NC) to calculate the correlation coefficient and *P* value.

### Statistical Analysis

The main aim of this study was to identify serum biomarkers that are associated with all-cause mortality in both day 1 and day 8 cohorts. Patients in each cohort were classified into tertiles based on baseline biomarker measurements. A total of 4 proteins at day 1 and 3 proteins at day 8 of randomization were analyzed by tertile levels. These included FGF23, tissue plasminogen activator (tPA), matrix metalloproteinase-8 (MMP-8), soluble urokinase plasminogen activator receptor (suPAR), and IL-6. Continuous variables were expressed as mean ± SD or median and interquartile range, and categorical variables were expressed as the frequencies and percentages. When analyzed as a continuous variable, FGF23 was naturally log-transformed to follow a normal distribution. In both cohorts, we estimated patient survival probabilities in tertiles for each of the 4 serum proteins using the Kaplan-Meier method and evaluated the survival differences across tertiles using the log-rank test. We performed Cox regression analyses to examine the relationships between serum levels of each protein and all-cause mortality, without and with adjustment for age, sex, congestive heart failure (CHF), SOFA score, and diabetic status. The lower tertile level for every protein was considered as the reference group. The relationships of tPA, MMP-8, suPAR, and IL-6 with all-cause mortality were analyzed in similar fashion to that used for FGF23. Reported *P* values were not adjusted for multiple comparisons. All the statistical analyses were performed using the SAS software version 9.4 (SAS Institute, Cary, NC).

## Results

### Patient Characteristics

**Figure 1:**
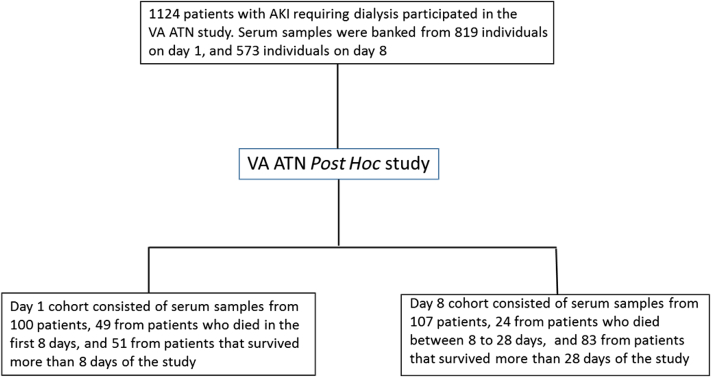
Flow diagram of serum samples analyzed in our substudy of the ATN Trial. AKI, acute kidney injury; VA ATN, Veteran’s Affairs Acute Renal Failure Trial Network

We used a total of 100 samples obtained on day 1 and 107 samples obtained on day 8 after randomization, as shown in Figure 1. Table 1 shows baseline characteristics of the patients who contributed samples to these analyses, stratified by mortality. Among the 100 patients in the day 1 cohort, 49 (49%) died by day 8. Patients who died had similar characteristics as those who survived >8 days. Among the 107 patients included in the day 8 sample cohort, 24 (22.4%) died by day 28. Characteristics of patients who died or survived to day 28 were similar, other than the percentage with a cardiovascular component of the SOFA score >2 (87.5% vs. 55.4%; *P* = 0.009).

**Table 1:** Characteristics of day 1 and day 8 patient cohorts stratified by survival status

Day 1 cohort				
Characteristic	Overall (*n* = 100)	Alive at day 8 (*n* = 51)	Died within first 8 days (*n* = 49)	*P* value^a^
Age (yr)	61.6 ± 14.5	62.1 ± 14.1	61.0 ± 15.0	0.73
Male	77 (77.0)	40 (78.4)	37 (75.5)	0.73
Congestive heart failure^b^	18 (19.1)	11 (23.4)	7 (14.9)	0.29
Diabetes mellitus^c^	31 (32.3)	16 (33.3)	15 (31.2)	0.83
CV-SOFA ≥2	62 (62.0)	28 (54.9)	34 (69.4)	0.14
Serum calcium (mg/dl)	7.64 ± 0.94	7.66 ± 0.92	7.61 ± 0.98	0.81
Serum PO4 (mg/dl)	5.63 ± 1.74	5.53 ± 1.79	5.75 ± 1.69	0.55

Day 8 cohort				
Characteristic	Overall (*n* = 107)	Alive at day 28 (*n* = 83)	Died between day 8 and day 28 (*n* = 24)	*P* value^a^
Age (yr)	60.4 ± 15.0	60.8 ± 14.5	58.8 ± 16.6	0.57
Male	74 (69.2)	54 (65.1)	20 (83.3)	0.15
Congestive heart failure^d^	24 (23.1)	17 (21.0)	7 (30.4)	0.34
Diabetes mellitus^e^	35 (33.3)	28 (34.1)	7 (30.4)	0.74
CV-SOFA ≥2	67 (62.6)	46 (55.4)	21 (87.5)	0.009
Serum calcium (mg/dl)	7.68 ± 0.93	7.76 ± 0.93	7.44 ± 0.90	0.15
Serum PO4 (mg/dl)	5.64 ± 1.88	5.48 ± 1.88	6.28 ± 1.77	0.096

CV-SOFA, coefficient of variation−sequential organ failure assessment; PO4, phosphate.

Data are presented as mean ± SD or no. (%).

a *P* values comparing surviving and deceased patients.

b Data missing on 6 patients (4 alive, 2 dead).

c Data missing on 4 patients (3 alive, 1 dead).

d Data missing on 3 patients (2 alive, 1 dead).

e Data missing on 2 patients (1 alive, 1 dead).

### SOMAscan Profile of Day 1 Samples Stratified by Mortality

Technical evaluation and validation of the SOMAscan assay have been standardized by SomaLogic, Inc. and confirmed by other independent studies.^11^ To detect protein abundance differences, a 1.2-fold cutoff and *P* < 0.05 have been used in recent studies.^22^ To validate these criteria, we randomly assigned each of the 2 QC samples to 2 separate groups for technical quantification validation of the 1305 proteins. Because the 2 QC samples were the replicates from the same healthy volunteer in each of the 9 assays performed, as expected, no differential proteins (false positive identifications) were detected between the 2 QC groups when a combination of a 1.2-fold cutoff and *P* < 0.05 were applied, whereas 3 and 23 proteins were detected to be a quantitatively differential when either a 1.2-fold cutoff or *P* < 0.05 was applied alone. A previous study showed that a combination of fold change and *P* value cutoff filtering resulted in more reproducible and reliable differential gene expression data.^24^ From the SOMAscan analysis of the AKI-D samples, we determined that serum levels of 33 proteins were higher, and 21 proteins were lower (fold change ≥1.2 and *P* < 0.05) in day 1 samples among AKI-D patients who died within the first 8 days compared with those who survived >8 days. The list of these 54 proteins is provided in Supplementary Table S1. As shown in Supplementary Table S1, none of the 54 proteins remained statistically significant after false discovery rate (FDR) multiple testing adjustment of *P* values. A recent study^25^ made an interactive web-based tool available to query results on individual SOMAmers by providing performance statistics such as interplate median coefficient of variation (CV), RFUs, fold-change probability distributions for intra- and interplate replicates, and critical value distributions for sets of intra- and interplate replicates. Using this resource, we found that an intraplate CV for FGF23 was 2.6%, with a total CV of 7.4%. From the data using this on-line tool, a 1.2-fold difference in abundance of FGF23 was associated with a *P* < 0.05. The assay variation for other biomarkers discussed in the following was also low, with median CV values of 5.3%, 4%, 5.4%, and 6.2% for tPA, MMP-8, suPAR, and IL-6, respectively.^25^

**Figure 2:**
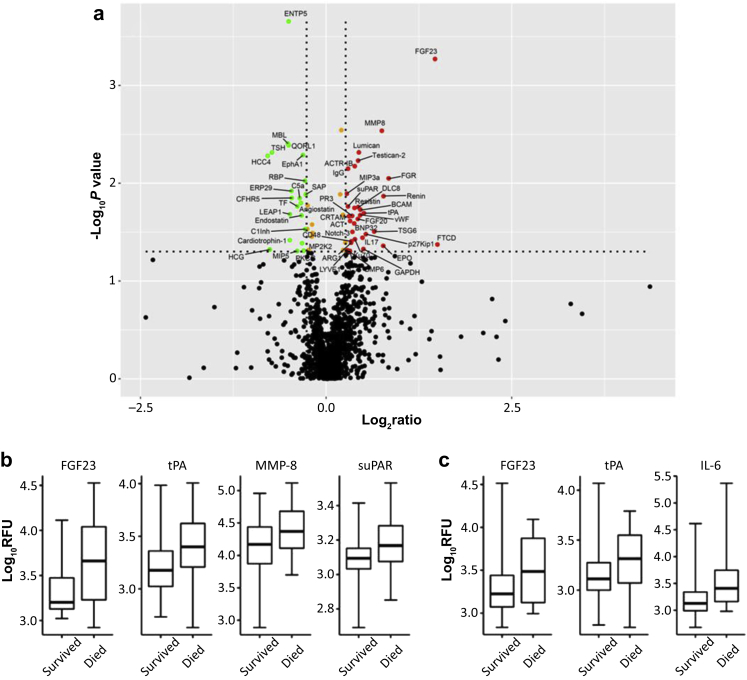
Changes in serum protein levels in samples obtained at day 1 from patients who died in < 8 days compared with patients who survived >8 days. (a) The volcano plot is used for visualizing of the relationship between fold changes and statistical significance. The vertical lines correspond to a 1.2-fold change in up-and-down expression, whereas the horizontal line represents a *P* value of 0.05. The black dots represent proteins with no statistically different fold change <1.2 and *P* ≥ 0.05; the red dots represent upregulated proteins with a fold change ≥1.2 and *P* < 0.05; green dots represent downregulated proteins with a fold change ≥1.2 and *P* < 0.05; and the yellow dots represent proteins with a fold change <1.2 and *P* < 0.05. (b) Box plots showing increased serum levels of fibroblast growth factor 23 (FGF23), tissue plasminogen activator (tPA), matrix metalloproteinase-8 (MMP8), and soluble urokinase plasminogen activator receptor (suPAR) in the patients who died in the first 8 days compared with those who survived >8 days (*P* = 0.0005, 0.020, 0.003, and 0.017, respectively) in the day 1 patient cohort. (c) Box plots showing increased serum levels of FGF23, tPA, and interleukin-6 (IL-6) in the patients who died between 8 and 28 days compared with those who survived beyond 28 days (*P* = 0.037, 0.037, 0.0049, respectively) in the day 8 patient cohort. Edges of boxes denote 25th and 75th percentiles, lines are median values, and error bars are minimum and maximum values. Slow off-rate modified aptamers scan relative fluorescence units (RFUs) were log transformed.

A volcano plot shown in Figure 2a demonstrates fold changes and the statistical significance of those proteins that were higher or lower in serum samples from day 1 among patients who died by day 8. The right upper hand corner of the volcano plot shows an isolated red dot corresponding to FGF23, which had a −log10 *P* value of >3 and was one of the proteins with a high fold-difference between patients who died and those who survived to day 8. Most serum proteins profiled in every sample (>1000 proteins), shown as black dots in the lower part of the volcano plot, did not show a significant statistical difference between those who died and those who survived.

When we compared the day 1 serum levels of the proteins from patients who died in <8 days with the day 8 serum levels from patients who died between 8 and 28 days, we found that only a few proteins were elevated at both day 1 and day 8. FGF23 and tPA were 2 of the proteins that were found elevated at both day 1 and day 8, and by our statistical analysis, they were both associated with high mortality. Supplementary Table S1 shows the SOMAscan values for 54 proteins that changed at day 1 and their values at day 8.

### IPA Analysis of Proteins Increased at Day 1 of the Patient Cohort Confirms Changes in Inflammation Associated With IL-6 Pathway

**Figure 3:**
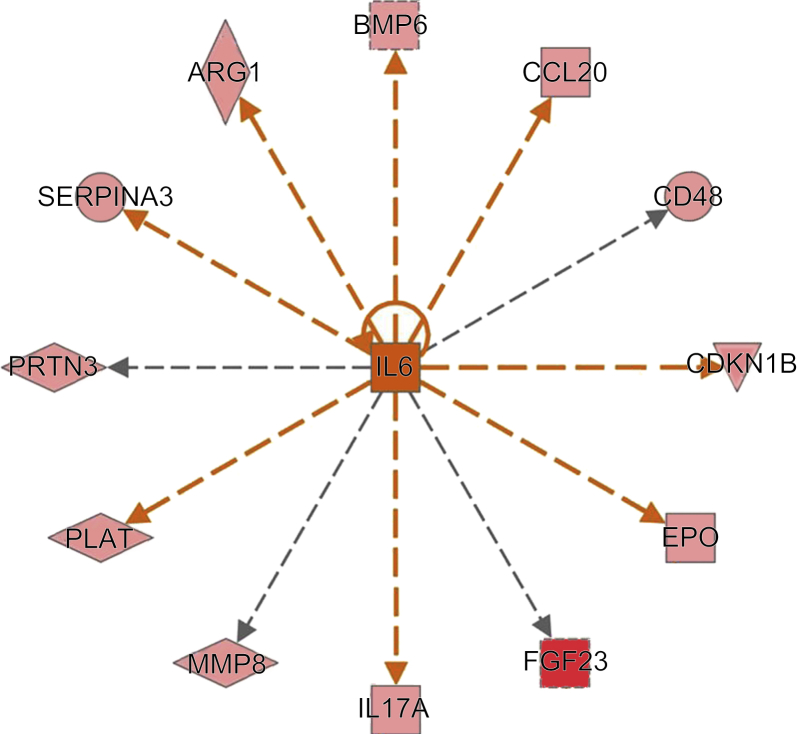
Predicted network of interleukin-6 (IL-6). IL-6 was identified using ingenuity pathway analysis as one of the top upstream regulators, shown here as being activated (*z*-score = 2.741 and *P* = 3.45E-10) in the day 1 patient cohort. Dashed orange lines denote that the IL-6 regulated downstream genes are predicted to be in the activated state and are in concordance to the increased serum levels in the patients who died within the first 8 days. Dashed gray lines indicate that the genes are regulated by IL-6, but the effects (activation or inhibition) are not predicted. The serum levels of all the proteins shown here were increased in patients who died in the first 8 days.

IPA identified top biological functions that changed in those patients with early mortality, including proteins associated with cellular movement of phagocytes, immune cell trafficking, inflammatory response, cell death, and survival. From a total of 33 serum proteins that were found elevated in AKI-D patients who died before day 8, a total of 10 proteins were associated with increased inflammation, and they included FGF23, MMP-8, bone morphogenetic protein-6, CC-motif chemokine 20, CD48 antigen, cyclin-dependent kinase inhibitor 1B, erythropoietin, IL-17A, α-1 antichimotrypsin, and arginase 1. As shown in Figure 3, IL-6 was identified as 1 of the top upstream regulators of these 10 proteins. In addition to increased inflammation, IPA demonstrated that increased expression of 3 serum proteins associated with increased coagulation in patients who died early (< 8 days). These included suPAR, von Willebrand factor, and tPA. Box plots in Figure 2b show the differences in SOMAscan intensities of the proteins associated with increased inflammation or coagulation (e.g., FGF23, tPA, MMP-8, and suPAR). The mean SOMAscan intensities of FGF23, tPA, MMP-8, and suPAR were 2822, 2213, 20,636, and 1313 RFUs, respectively, in the patients who survived >8 days. FGF23 levels were 2.76-fold higher (*P* = 0.0005), tPA levels were 1.42-fold higher (*P* = 0.020), MMP-8 levels were 1.68-fold higher (*P* = 0.003), and suPAR levels were 1.23-fold higher (*P* = 0.017) among patients who died by day 8 compared with patients who survived beyond day 8.

### SOMAscan Profile of Day 8 Samples Stratified by Mortality

We performed SOMAscan profiles of serum samples obtained from 107 patients on day 8 after randomization. Figure 2c shows box plots corresponding to the SOMAscan intensities for 3 proteins with higher levels in serum from patients who died between 8 and 28 days compared with patients who survived >28 days. FGF23 levels were 1.4-fold higher (*P* = 0.037), tPA levels were 1.38-fold higher (*P* = 0.037), and IL-6 levels were 5.81-fold higher (*P* = 0.0049). We showed that higher levels of FGF23, tPA, MMP-8, suPAR, and IL-6, as measured by SOMAscan, were correlated with increased mortality, and these proteins were associated with increased inflammation and coagulation.

### ELISA Validation of FGF23 Levels Measured by SOMAscan

**Figure 4:**
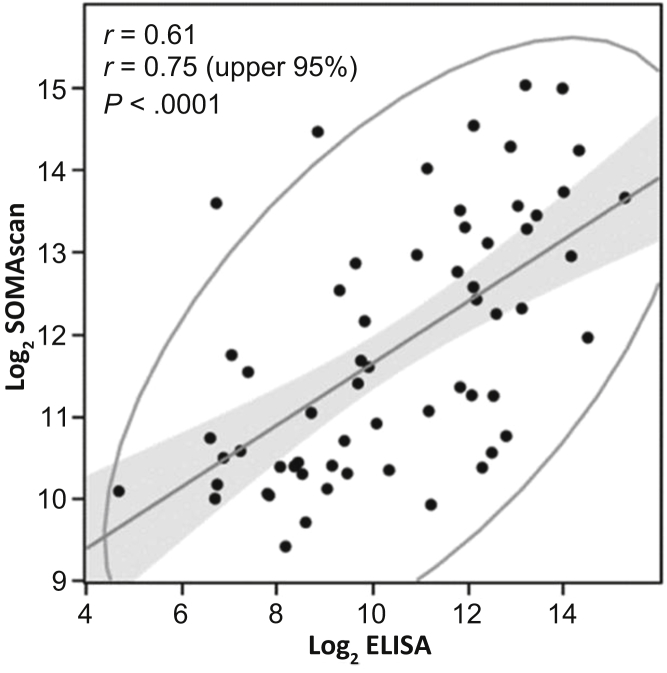
Correlation plot between Log2 slow off-rate modified aptamers (SOMA) scan values and Log2 C-terminus enzyme-linked immunosorbent assay (ELISA) for serum fibroblast growth factor 23 (FGF23) levels. The ellipse is for confidence coefficient of 95%, within which 95% of the samples (points) were enclosed. The correlation fit line is also shown.

To further validate the FGF23 serum levels measured by SOMAscan, we performed ELISA analysis of FGF23. SOMAscan intensities correlated positively with ELISA intensities with a correlation coefficient of 0.61 for all of the 61 samples analyzed, and a correlation coefficient of 0.75 for the upper 95% of samples (Figure 4). This correlation was statistically significant (*P* < 0.0001).

### Association of Day 1 FGF23, tPA, MMP-8, and suPAR Levels With Mortality by Day 8

**Figure 5:**
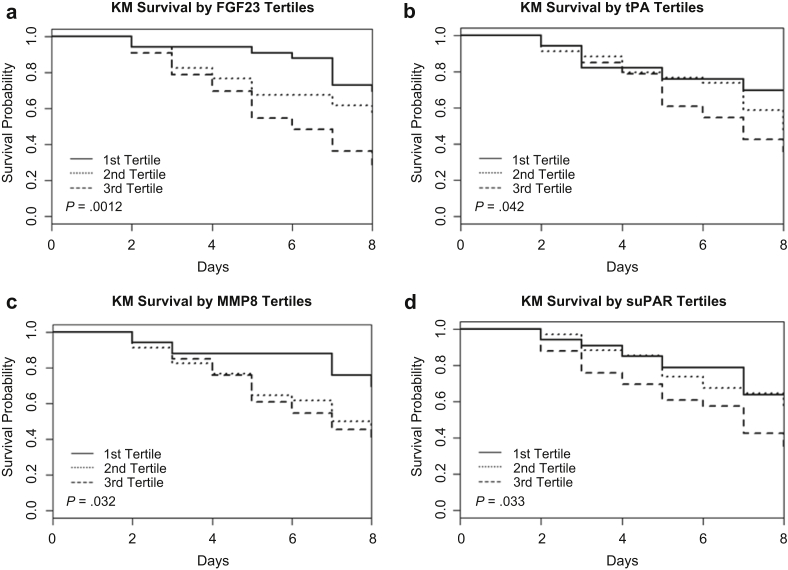
Day 1 cohort event-free survival stratified by biomarker tertiles. (a) Fibroblast growth factor 23 (FGF23), (b) tissue plasminogen activator (tPA), (c) matrix metalloproteinase-8 (MMP8), and (d) soluble urokinase plasminogen activator receptor (suPAR). KM, Kaplan-Meier.

We analyzed patient mortality by day 8 stratified by tertiles of FGF23, tPA, MMP-8, and suPAR levels (Table 2 and Figure 5). Characteristics of the day 1 patient cohorts stratified by FGF23 tertiles are presented in Supplementary Table S2. Patients with the highest tertile of levels for each of FGF23, tPA, MMP-8, and suPAR had higher mortality on univariate analysis compared with the patients in the lowest tertile for each. On multivariate analysis of mortality risk in the highest tertile, FGF23, tPA, and MMP-8 remained significant. When considered as a continuous variable, after covariate adjustment, higher FGF23 levels were also significantly associated with mortality, with the risk of death nearly doubled for each 1-unit increment of log-adjusted FGF23 levels (hazard ratio [HR]: 1.91; 95% confidence interval [CI]: 1.39−2.63; *P* < 0.001).

**Table 2:** Risk (hazard ratio and 95% confidence interval) of death within 8 days by tertiles of 4 serum biomarkers^a^

Protein	*N*	Median value (IQR)	Univariate		Multivariate	
			HR (95% CI)	*P* value	HR (95% CI)	*P* value
**Day 1**						
FGF23						
Tertile 1	33	1298 (1149−1390)	1	—	1	—
Tertile 2	34	2146 (1833−2355)	1.71 (0.77−3.81)	0.770	1.78 (0.80−3.95)	0.1643
Tertile 3	33	8849 (5154−12,990)	3.35 (1.60−7.04)	0.0014	3.46 (1.57−7.61)	0.002
tPA						
Tertile 1	33	1080 (850−1246)	1	—	1	—
Tertile 2	34	1928 (1785−2188)	1.80 (0.83−3.91)	0.1341	1.78 (0.80−3.95)	0.1521
Teritle 3	33	4185 (3480−6429)	2.46 (1.15−5.24)	0.0191	2.71 (1.26−5.84)	0.0106
MMP-8						
Tertile 1	33	5139 (3844−8268)	1	—	1	—
Tertile 2	34	14,427 (12,902−21,521)	2.34 (1.09−5.02)	0.0277	2.27 (1.03−5.01)	0.0416
Tertile 3	33	44,517 (33,901−64,913)	2.38 (1.10−5.13)	0.0268	2.74 (1.25−6.01)	0.0117
suPAR						
Tertile 1	33	1045 (965−1133)	1	—	1	—
Tertile 2	34	1419 (1338−1482)	1.26 (0.59−2.70)	0.5462	1.22 (0.54−2.75)	0.6309
Tertile 3	33	2231 (2013−2599)	2.24 (1.11−4.54)	0.0244	1.96 (0.94−4.08**)**	0.07

CI, confidence interval; HR, hazard ratio; IQR, interquartile range.

Age, sex, congestive heart failure, and presence of diabetes were included for multivariate analysis.

a Fibroblast growth factor 23 (FGF23), tissue plasminogen activator (tPA), neutrophil collagenase (matrix metalloproteinase-8 [MMP8]), and soluble urokinase plasminogen activator receptor (suPAR), which were found elevated at day 1 by slow off-rate modified aptamers scan in samples from patients with acute kidney injury who required dialysis and died within 8 days.

### Association of Day 8 FGF23, tPA, and IL-6 With Mortality by Day 28

**Figure 6:**
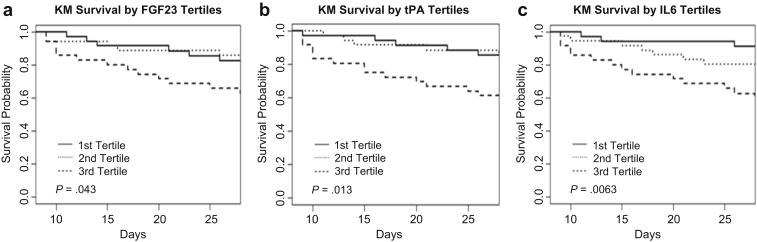
Day 8 cohort event-free survival stratified by biomarker tertiles. (a) Fibroblast growth factor 23 (FGF23), (b) tissue plasminogen activator (tPA), and (c) interleukin-6 (IL-6). KM, Kaplan-Meier.

In the cohort of patients who provided day 8 samples, we analyzed patient mortality by day 28 stratified by tertiles of FGF23, tPA, and IL-6 levels (Table 3 and Figure 6). Characteristics of the day 8 patient cohorts stratified by the FGF23 tertile are presented in Supplementary Table S2. Patients with the highest tertile levels for each of FGF23, tPA, and IL-6 had higher mortality compared with the patients in the lowest tertile for each, with tPA and IL-6 remaining significant after multivariate adjustment. In an unadjusted analysis, FGF23 analyzed as a continuous variable suggested that 1-unit increments in FGF23 in natural log-scale were associated with a 50% increased hazard of death (HR: 1.50; 95% CI: 1.02−2.21; *P* = 0.04). However, after covariate adjustment, FGF23 risk became insignificant (HR: 1.37; 95% CI: 0.92−2.05; *P* = 0.12), possibly due to a nonlinear relationship between mortality risk and FGF23.

**Table 3:** Risk (hazard ratio and 95% confidence interval) of death from 8 to 28 days by tertiles of 3 serum biomarkers^a^

Protein	*N*	Median value (IQR)	Univariate		Multivariate	
			HR (95% CI)	*P* value	HR (95% CI)	*P* value
**Day 8**						
FGF23						
Tertile 1	35	1108 (1026−1239)	1	—	1	—
Tertile 2	36	1884 (1518−2098)	0.82 (0.25−2.70)	0.7491	0.94 (0.28−3.13)	0.9288
Tertile 3	36	6064 (3380−11,161)	2.42 (0.92−6.37)	0.0730	2.42 (0.90−6.51)	0.0785
tPA						
Tertile 1	35	895 (842−1057)	1	—	1	—
Tertile 2	36	1395 (1259−1629)	0.99 (0.28−3.44)	0.9966	0.83 (0.21−3.22)	0.7950
Teritle 3	36	3183 (2473−4457)	3.15 (1.13−8.76)	0.0276	5.21 (1.67−16.21)	0.0043
IL-6						
Tertile 1	35	952 (822−1096)	1	—	1	—
Tertile 2	36	1405 (1302−1590)	2.32 (0.60−8.98)	0.221	2.64 (0.66−10.49)	0.1671
Teritle 3	36	3518 (2585−6131)	5.50 (1.55−8.83)	0.008	5.93 (1.54−22.82)	0.0095

CI, confidence interval; HR, hazard ratio; IQR, interquartile range.

*P* < 0.05 significant: FGF23, tPA, and IL-6.

a Fibroblast growth factor 23 (FGF23), tissue plasminogen activator (tPA), and interleukin 6 (IL-6) found elevated by slow off-rate modified aptamers scan in samples from patients with acute kidney injury who required dialysis obtained at day 8 of randomization.

## Discussion

This is the first study that used SOMAscan to examine the proteome of serum samples from AKI-D patients. In this *post hoc* analysis of serum samples obtained within 24 hours after enrollment in the VA/NIH ATN study, we found that: (i) plasma levels of 33 proteins were increased in patients who died in <8 days compared with patients who survived >8 days; (ii) among those 33 proteins, high levels of FGF23, tPA, MMP-8, and suPAR were strongly associated with increased mortality; (iii) IPA showed that most of the proteins associated with early mortality were involved in distinct molecular pathways, including increased systemic inflammation, increased coagulation, and increased endothelial cell injury; and (iv) SOMAscan analysis of serum samples obtained at day 8 after study enrollment further corroborated that high serum levels of FGF23, tPA, and IL-6 were also associated with increased mortality between 8 and 28 days.

Availability and examination of serum samples at the 2 separate timepoints of study initiation (day 1 and day 8) allowed investigation of inflammatory mechanisms associated with phases of acute injury and repair.^26^, ^27^ Identifying injury and/or repair markers that define early mortality in AKI-D patients and comparing them with injury and/or repair markers in those who survived beyond the acute phase of injury could provide further insight into mechanistic pathways in human AKI. Previous studies assessed increased mortality after AKI ranging from 28 days to 6 months. However, a recent study used a piecewise survival model and the data from ATN study to analyze survival after an episode of AKI that required dialysis.^28^ That study provided better definition of the natural history of survival after an episode of AKI and demonstrated that approximately one-half of the patients died during the first 8 days in the hyperacute and acute phases of AKI. We performed proteomic analysis of samples obtained after 24 hours, or the hyperacute phase, of enrollment in the ATN study, in which the incidence of mortality was 49% in first 8 days, and performed similar proteomic analysis on samples obtained at day 8, or the so-called acute phase, during which mortality was 22.4% in first 28 days. This type of analysis allowed us to investigate how biomarkers and molecular mechanisms evolve during AKI.

We found that high serum FGF23 levels were associated with increased mortality in AKI-D patients. The abundance levels of FGF23 were confirmed by ELISA, with a positive correlation between SOMAscan and ELISA measurements. It should be noted that the ELISA assay targets the C-terminus of FGF23, whereas the SOMAscan assay measures the total FGF23 in serum, which could account for the imperfect correlation between the 2 assays. FGF23 is a major regulator of serum phosphate and calcium homeostasis. In CKD patients, circulating FGF23 gradually increases with declining renal function to maintain normal serum phosphate.^29^ Also, several studies showed a strong association between serum FGF23 levels and an increased risk of cardiovascular events and mortality in CKD patients.^30^, ^31^, ^32^ FGF23 circulates as both an intact molecule and carboxy-terminal fragments generated through cleavage by proprotein convertases.^33^ Recent studies demonstrated a correlation between increased serum FGF23 levels and increased morbidity and mortality rates in AKI patients who underwent cardiac surgery.^34^ Another study reported high levels of plasma C-terminal FGF23 after cardiac bypass surgery, with development of AKI, need for renal replacement, and death.^35^ Higher urinary and plasma FGF23 levels were also found in patients admitted to the intensive care unit, and high serum FGF23 levels were correlated with increased incidence of AKI and/or death.^36^ FGF23 is also linked to iron metabolism, erythropoiesis, inflammation, insulin resistance, proteinuria, and left ventricular hypertrophy.^37^ Recent studies showed that high serum FGF23 levels inhibited neutrophil function during CKD.^38^, ^39^

Our study was the first to report an association of increased serum tPA levels with increased mortality in AKI-D patients. tPA, which is a member of the serine protease family, is a major fibrinolytic agent involved in recruitment of inflammatory cells. Other functions of tPA include turnover of extracellular matrix components through activation of MMPs and immunomodulatory functions.^40^, ^41^ Previous studies in animal models reported upregulation of tPA expression in proximal tubular epithelial cells of ischemic kidneys. In addition, elimination of tPA by antisense treatment reduced neutrophil influx and protected renal function during ischemia−reperfusion injury, which suggested inhibition of tPA as a novel strategy to ameliorate ischemic AKI.^42^ Several other studies also suggested that tPA participates in the process of kidney fibrosis that leads to progression of CKD.^43^, ^44^, ^45^, ^46^

This study demonstrated, for the first time, that high serum levels of MMP-8 were associated with increased mortality. MMP-8 expression is induced by various inflammatory cytokines, including IL-1β, tumor necrosis factor-α, and CD40 ligand.^47^, ^48^ Several animal and human studies demonstrated an important role of MMP-8 in the pathogenesis of atherosclerosis. Gene targeting studies also demonstrated reduced atherosclerosis development in MMP8 knockout mice.^49^ A multicenter cohort study demonstrated increased serum MMP-8 levels in septic patients admitted to the ICU.^50^

To our knowledge, this was the first study to show that high serum levels of suPAR were associated with increased mortality in AKI-D patients. suPAR plays a role in immune responses, such as cell adhesion, migration, chemotaxis, proteolysis, immune activation, signal transduction, and tissue remodeling. A recent meta-analysis study showed that high suPAR levels were associated with mortality in septic patients.^51^

Although IL-6 serum levels at day 1 were elevated in AKI patients who died early, there was no statistical significance as demonstrated with serum levels of FGF23, tPA, MMP8, or suPAR. However, serum IL-6 levels were 5.8-fold higher in patients who died between 8 and 28 days when measured on day 8 samples. Also, the highest tertile levels of serum IL-6 were correlated with increased mortality. Recent studies in several animal models showed a close correlation between IL-6 expression and AKI.^52^, ^53^ Kidney resident cells, including podocytes, endothelial cells, mesangial cells, and tubular epithelial cells can secrete IL-6. A study showed that after leukocytes infiltrated the injured kidney in a model of ischemia–reperfusion injury, they produced maladaptive IL-6 when their TLR4 receptors interacted with HMGB1 released by injured renal cells.^54^ Previous studies showed that the proinflammatory cytokines IL-6 and IL-8 were elevated early in AKI patients and were associated with prolonged mechanical ventilation.^55^

Our study had certain limitations. First, we only had serum samples at day 1 and day 8 of randomization, with secondary data analyses of a sample of the clinical trial study population. However, we addressed these shortcomings by using statistical methods to attenuate potential biases. Second, to address the issue of false positive identifications, we used both fold change and *P* value cutoff filtering to obtain a list of proteins with a differential abundance in serum, which was more reliable than using a fold change or *P* value cutoff alone.^24^ However, because of the number of target aptamers used in the SOMAscan assay, we were unable to exclude the possibility of false discovery through adjustment for multiple comparisons due to the limited number of samples analyzed. Exclusion of false discovery will require a substantially larger cohort of samples. With that caveat, the observed 2.76-fold elevation in FGF23 was associated with a nominal *P* value of 0.0005. Furthermore, some of the biomarkers identified in this study might only be applicable for specific subtypes of AKI or mortality subgroups. The novel biomarkers discovered in our study warrants further independent validation for high mortality in AKI-D patients.

In conclusion, our study used a high throughput proteomic technology to identify novel biomarkers of mortality and defined biological pathways associated with severe AKI-D.

## Disclosure

All the authors declared no competing interests.

## Supplementary Material

Table S1 Comparison between the serum levels of proteins that changed significantly in the day 1 and day 8 cohorts.

Table S2 Patient characteristics of day 1 and day 8 cohorts stratified by serum fibroblast growth factor 23 (FGF23) tertiles.
