# Supplementary material for: Aptamer-Based Proteomics Identifies Mortality-Associated Serum Biomarkers in Dialysis-Dependent AKI Patients
Source: Kidney Int Rep. 2018 May 3;3(5):1202–13. doi: 10.1016/j.ekir.2018.04.012 (PMC6127416; doi:10.1016/j.ekir.2018.04.012)
Supplement: Table S1 — Comparison between the serum levels of proteins that changed significantly in the day 1 and day 8 cohorts. [file mmc1.pdf]

Supplemental Table 1 Proteins with significant changes in serum levels (1.2-fold and *p* <0.05) as measured by SOMAscan assays in the patients died (D) in the first 8 days as compared to those who survived (S) >8 days in the Day 1 cohort. Their serum levels in the patients died (D) between 8-28 days as compared to those who survived (S) >28 days in the Day 8 cohort are also listed

				Day 1							Day 8						
**UniProt**	**Gene**	**Short Portein Name**	**Protein Name**	**Mean (S)**	**SD (S)**	**Mean (D)**	**SD (D)**	**Ratio (D/S)**	***p*-value**	**FDR**	**Mean (S)**	**SD (S)**	**Mean (D)**	**SD (D)**	**Ratio (D/S)**	***p*-value**	**FDR**
O95954	FTCD	FTCD	Formimidoyltransferase-cyclodeaminase	13262	21380	37525	105398	2.83	0.0425	0.9683	13762	27990	10948	15816	0.80	0.7184	0.8920
Q9GZV9	FGF23	FGF23	Fibroblast growth factor 23	2822	2790	7799	8027	2.76	0.0005	0.3498	3446	5722	4834	4042	1.40	0.0366	0.3094
P09769	FGR	FGR	Tyrosine-protein kinase Fgr	747	1230	1341	2065	1.80	0.0089	0.7902	783	711	849	854	1.08	0.7796	0.9215
P00797	REN	Renin	Renin	3313	3329	5665	5338	1.71	0.0135	0.7902	2125	2390	3008	3039	1.42	0.0191	0.2800
P01588	EPO	Epo	Erythropoietin	6590	12277	11241	16665	1.71	0.0437	0.9321	6481	13009	3921	2561	0.60	0.8911	0.9706
P22894	MMP8	MMP-8	Neutrophil collagenase	20636	19068	34730	29872	1.68	0.0029	0.6861	15573	16264	14210	10969	0.91	0.8182	0.9418
P98066	TNFAIP6	TSG-6	Tumor necrosis factor-inducible gene 6 protein	2139	1147	3355	3313	1.57	0.0312	0.9321	1801	890	2351	1646	1.31	0.4042	0.7016
P46527	CDKN1B	p27Kip1	Cyclin-dependent kinase inhibitor 1B	1959	623	2842	2602	1.45	0.0331	0.9400	2661	3795	2825	1634	1.06	0.1489	0.4969
P00750	PLAT	tPA	Tissue-type plasminogen activator	2213	2089	3150	2166	1.42	0.0202	0.7902	1840	1719	2544	1625	1.38	0.0367	0.3094
P04406	GAPDH	GAPDH	Glyceraldehyde-3-phosphate dehydrogenase	35717	49337	50649	75314	1.42	0.0471	0.9683	35617	57912	37557	41390	1.05	0.9104	0.9722
Q16552	IL17A	IL-17	Interleukin-17A	684	337	962	978	1.41	0.0362	0.9683	746	275	904	628	1.21	0.4321	0.7152
P50895	BCAM	BCAM	Basal cell adhesion molecule	1759	535	2425	1869	1.38	0.0186	0.7902	1686	452	2077	1185	1.23	0.1555	0.5074
P04275	VWF	vWF	von Willebrand factor	129593	66356	178237	92646	1.38	0.0212	0.7902	125948	62370	143070	67315	1.14	0.4850	0.7517
P51884	LUM	Lumican	Lumican	940	378	1277	672	1.36	0.0048	0.6861	837	277	912	259	1.09	0.1406	0.4880
P63167	DYNLL1	DLC8	Dynein light chain 1, cytoplasmic	1067	1592	1440	1396	1.35	0.0175	0.7902	955	556	1656	2267	1.73	0.2338	0.5799
Q92563	SPOCK2	Testican-2	Testican-2	658	223	887	390	1.35	0.0059	0.6976	641	320	710	275	1.11	0.1583	0.5097
Q9NP95	FGF20	FGF-20	Fibroblast growth factor 20	570	346	766	593	1.34	0.0231	0.8156	539	328	551	165	1.02	0.3384	0.6542
P12956	XRCC6	Ku70	X-ray repair cross-complementing protein 6	1173	731	1535	1184	1.31	0.0374	0.9683	1101	718	1254	806	1.14	0.3046	0.6300
P36896	ACVR1B	ACTR-IB	Activin receptor type-1B	766	321	1002	654	1.31	0.0067	0.7132	893	394	868	242	0.97	0.9823	0.9908
Q9HD89	RETN	Resistin	Resistin	11204	6910	14609	7080	1.30	0.0179	0.7902	8402	5585	10414	6840	1.24	0.1727	0.5212
P16860	NPPB	BNP-32	Brain natriuretic peptide 32	1301	474	1693	1049	1.30	0.0258	0.8629	1544	750	1635	637	1.06	0.3434	0.6591
Q9UM47	NOTCH3	Notch-3	Neurogenic locus notch homolog protein 3	564	339	722	486	1.28	0.0314	0.9321	533	261	574	262	1.08	0.4821	0.7501
P24158	PRTN3	PR-3	Myeloblastin	34571	18672	44143	19986	1.28	0.0215	0.7902	27841	20456	28916	19007	1.04	0.5779	0.8241
P09326	CD48	CD48	CD48 antigen	1124	401	1422	663	1.27	0.0392	0.9683	1200	483	1463	859	1.22	0.1339	0.4696
P05089	ARG1	ARG1	Arginase-1	3001	1879	3793	2127	1.26	0.0407	0.9683	3572	2046	4025	3760	1.13	0.9059	0.9715
P01011	SERPINA3	ACT	Alpha-1-antichymotrypsin	39467	20991	49397	23008	1.25	0.0243	0.8361	31178	15599	28272	12886	0.91	0.4402	0.7199
P10643	C7	C7	Complement component C7	2339	907	2926	1508	1.25	0.0493	0.9683	2288	979	2660	1256	1.16	0.2407	0.5812
P22004	BMP6	BMP-6	Bone morphogenetic protein 6	769	308	954	440	1.24	0.0498	0.9683	794	399	823	277	1.04	0.3796	0.6833
O95727	CRTAM	CRTAM	Cytotoxic and regulatory T-cell molecule	1332	226	1650	917	1.24	0.0218	0.7902	1436	649	1344	418	0.94	0.4539	0.7294
P01857	IGHG1	IgG	Immunoglobulin G	172792	51665	212127	71569	1.23	0.0071	0.7132	167470	43028	189824	54964	1.13	0.0968	0.4137
Q03405	PLAUR	suPAR	Urokinase plasminogen activator surface receptor	1313	385	1611	601	1.23	0.0172	0.7902	1391	405	1476	414	1.06	0.4244	0.7142
Q9Y5Y7	LYVE1	LYVE1	Lymphatic vessel endothelial hyaluronic acid receptor 1	653	349	797	347	1.22	0.0484	0.9683	643	258	742	363	1.15	0.2504	0.5943
P78556	CCL20	MIP-3a	C-C motif chemokine 20	499	147	605	246	1.21	0.0128	0.7902	514	188	550	176	1.07	0.2574	0.5972
P02743	APCS	SAP	Serum amyloid P-component	30481	9609	25198	10472	0.83	0.0130	0.7902	25963	7521	23886	7321	0.92	0.2410	0.5812
P05155	SERPING1	C1Inh	Plasma protease C1 inhibitor	9662	4545	7956	2598	0.82	0.0295	0.9153	10212	4128	9827	4352	0.96	0.5868	0.8241
P02753	RBP4	RBP	Retinol-binding protein 4	217	87	178	59	0.82	0.0095	0.7902	263	65	250	68	0.95	0.3471	0.6640
P05129	PRKCG	PKC-G	Protein kinase C gamma type	726	516	589	192	0.81	0.0491	0.9683	712	469	966	1182	1.36	0.3854	0.6872
P21709	EPHA1	EphA1	Ephrin type-A receptor 1	1716	542	1382	626	0.81	0.0052	0.6861	2040	634	1919	545	0.94	0.4633	0.7353
P36507	MAP2K2	MAPKK2	Dual specificity mitogen-activated protein kinase kinase 2	3291	1870	2628	1887	0.80	0.0411	0.9683	2944	1656	2263	1057	0.77	0.0583	0.3457

P39060	COL18A1	Endostatin	Endostatin	90752	37356	72123	27759	0.79	0.0214	0.7902	98131	43830	80994	39548	0.83	0.0849	0.3923
P00747	PLG	Angiostatin	Angiostatin/Plasminogen	7174	3395	5669	2936	0.79	0.0159	0.7902	8705	4132	7135	3565	0.82	0.0630	0.3552
P01031	C5	C5a	C5a anaphylatoxin	13212	5975	10330	6804	0.78	0.0142	0.7902	15316	6905	13796	8462	0.90	0.1828	0.5310
P13726	F3	TF	Tissue factor	4752	3784	3635	4768	0.76	0.0172	0.7902	5413	4125	5656	3347	1.04	0.8290	0.9423
Q16663	CCL15	MIP-5	C-C motif chemokine 15	9159	5753	6991	3902	0.76	0.0493	0.9683	8848	5411	9303	5570	1.05	0.8222	0.9423
Q9BXR6	CFHR5	FHR-5	Complement factor H-related protein 5	3811	2967	2764	1343	0.73	0.0141	0.7902	3327	1313	3096	1392	0.93	0.4091	0.7054
P30040	ERP29	ERp29	Endoplasmic reticulum resident protein 29	4301	2466	3112	2424	0.72	0.0120	0.7902	4466	2169	3779	1848	0.85	0.1376	0.4814
P81172	HAMP	LEAP-1	Hepcidin	35617	22630	25403	19985	0.71	0.0208	0.7902	24690	20433	19263	23272	0.78	0.0394	0.3094
Q16619	CTF1	CT-1	Cardiotrophin-1	1485	1990	1058	225	0.71	0.0381	0.9683	1527	812	1375	497	0.90	0.1968	0.5406
O75356	ENTPD5	ENTPD5	Ectonucleoside triphosphate diphosphohydrolase 5	3843	1586	2708	1232	0.70	0.0002	0.2890	3790	1124	3706	1983	0.98	0.3684	0.6787
O95825	CRYZL1	QORL1	Quinone oxidoreductase-like protein 1	3455	1767	2435	1034	0.70	0.0041	0.6861	4619	2004	5329	3185	1.15	0.3830	0.6849
P11226	MBL2	MBP-C	Mannose-binding protein C	20597	10281	14411	8195	0.70	0.0039	0.6861	22801	13979	20624	12676	0.90	0.5417	0.7984
P01222	TSHB	TSH-B	Thyroid stimulating hormone subunit beta	1197	864	722	464	0.60	0.0049	0.6861	3022	4569	2366	2455	0.78	0.4649	0.7353
P01215	CGA	HCG	Human chorionic gonadotropin	1662	2317	982	541	0.59	0.0477	0.9683	1613	2631	1303	1058	0.81	0.7311	0.8979
O15467	CCL16	HCC-4	C-C motif chemokine 16	38747	40283	22466	19988	0.58	0.0053	0.6861	36402	24267	30623	22686	0.84	0.1537	0.5064

FDR, Benjamini & Hochberg adjusted p-value
