# Supplementary material for: Aptamer-Based Proteomics Identifies Mortality-Associated Serum Biomarkers in Dialysis-Dependent AKI Patients
Source: Kidney Int Rep. 2018 May 3;3(5):1202–13. doi: 10.1016/j.ekir.2018.04.012 (PMC6127416; doi:10.1016/j.ekir.2018.04.012)
Supplement: Table S2 — Patient characteristics of day 1 and day 8 cohorts stratified by serum fibroblast growth factor 23 (FGF23) tertiles. [file mmc2.pdf]

Supplemental Table 2 Patient characteristics of day 1 and day 8 cohorts stratified by tertiles of serum fibroblast growth factor-23 (FGF23) measured by SOMAscan (RFU) (study population for day 1 cohort n=100, study population for day 8 cohort n=107)

Characteristics of day 1 Cohort	Overall	FGF23 Tertile 1	FGF23 Tertile 2	FGF23 Tertile 3
N	100	33	34	33
FGF23 RFU (median)		1298	2146	8849
Demographics				
Age, yr	62±14	66±14	61±14	58±15
Male	77	79	82	70
Congestive heart failure	19	19	16	23
Diabetes	32	32	36	28
CV-SOFA ≥ 2	62	55	62	70
Serum calcium (mg/dl)	7.6±0.9	7.4±0.8	7.8±0.9	7.6±1.1
Serum PO4 (mg/dl)	5.6±1.7	5.3±1.6	5.7±2.1	5.9±1.4

Characteristics of day 8 cohort	Overall	FGF23 Tertile 1	FGF23 Tertile 2	FGF23 Tertile 3
N	107	35	36	36
FGF23 RFU (median)		1108	1884	6064
Demographics				
Age, yr	60±15	59±16	61±13	61±16
Male	70	74	69	66
Congestive heart failure	23	24	15	29
Diabetes	33	26	29	44
CV-SOFA ≥ 2	63	49	60	80
Serum calcium (mg/dl)	7.7±0.9	7.5±1.0	7.6±0.9	7.9±0.9
Serum PO4 (mg/dl)	5.6±1.9	6.0±1.4	5.5±2.1	5.3±2.0

Continuous variables are expressed as mean ± SD, categorical variables as %.
